# Transcriptome and Molecular Pathway Analysis of the Hepatopancreas in the Pacific White Shrimp *Litopenaeus vannamei* under Chronic Low-Salinity Stress

**DOI:** 10.1371/journal.pone.0131503

**Published:** 2015-07-06

**Authors:** Ke Chen, Erchao Li, Tongyu Li, Chang Xu, Xiaodan Wang, Heizhao Lin, Jian G. Qin, Liqiao Chen

**Affiliations:** 1 Laboratory of Aquaculture Nutrition and Environmental Health, School of Life Sciences, East China Normal University, Shanghai, China; 2 Shenzhen Base of South China Sea Fisheries Research Institute, Shenzhen, China; 3 School of Biological Sciences, Flinders University, Adelaide, Australia; Chinese Academy of Fishery Sciences, CHINA

## Abstract

The Pacific white shrimp *Litopenaeus vannamei* is a euryhaline penaeid species that shows ontogenetic adaptations to salinity, with its larvae inhabiting oceanic environments and postlarvae and juveniles inhabiting estuaries and lagoons. Ontogenetic adaptations to salinity manifest in *L*. *vannamei* through strong hyper-osmoregulatory and hypo-osmoregulatory patterns and an ability to tolerate extremely low salinity levels. To understand this adaptive mechanism to salinity stress, RNA-seq was used to compare the transcriptomic response of *L*. *vannamei* to changes in salinity from 30 (control) to 3 practical salinity units (psu) for 8 weeks. In total, 26,034 genes were obtained from the hepatopancreas tissue of *L*. *vannamei* using the Illumina HiSeq 2000 system, and 855 genes showed significant changes in expression under salinity stress. Eighteen top Kyoto Encyclopedia of Genes and Genomes (KEGG) pathways were significantly involved in physiological responses, particularly in lipid metabolism, including fatty-acid biosynthesis, arachidonic acid metabolism and glycosphingolipid and glycosaminoglycan metabolism. Lipids or fatty acids can reduce osmotic stress in *L*. *vannamei* by providing additional energy or changing the membrane structure to allow osmoregulation in relevant organs, such as the gills. Steroid hormone biosynthesis and the phosphonate and phosphinate metabolism pathways were also involved in the adaptation of *L*. *vannamei* to low salinity, and the differential expression patterns of 20 randomly selected genes were validated by quantitative real-time PCR (qPCR). This study is the first report on the long-term adaptive transcriptomic response of *L*. *vannamei* to low salinity, and the results will further our understanding of the mechanisms underlying osmoregulation in euryhaline crustaceans.

## Introduction

Salinity is one of the main environmental factors that exert a selection pressure on aquatic organisms, and variations in ambient salinity can directly impact the composition and osmolality of body fluids in aquatic animals [1]. Aquatic crustaceans inhabit environments with varying salinities, such as freshwater to seawater, and a change of environment requires crustaceans to regulate hemolymph osmolytes via osmoregulation [2, 3]. Crustaceans display several patterns of osmoregulation, including osmoconformation, hyper-osmoregulation and hypo-osmoregulation [2, 4]. Studies have shown that salinity stress can reduce salt diffusion between hemolymph and the environment because water is absorbed from the medium, which leads to swollen cells [5]. When confronted with salinity stress, aquatic animals are forced to osmoregulate by altering the expression of various enzymes and transporters, and the physiological adaptations associated with such functional changes are energy intensive [6]. Therefore, it is important to determine the amount of energy that is required during adaptations to different salinities. In addition, although the biochemical osmoregulation mechanisms of crustaceans have been studied [1, 3, 4], the molecular adaptive mechanisms for energy mobilization are not known.

The Pacific white shrimp *Litopenaeus vannamei* is a typical euryhaline crustacean species that lives in coastal and oceanic environments, and its larvae develop in the ocean, whereas the postlarvae, juveniles and adults live in estuaries and lagoons [7]. A hyper-hypo-osmoregulation process exists in the life history of *L*. *vannamei*, and an adaptive mechanism must exist to cope with the environmental salinity fluctuation or long-term low salinity stress. Therefore, *L*. *vannamei* can serve as an animal model in the study of adaptive mechanisms in euryhaline crustacean to changes in salinity. Because *L*. *vannamei* is an important commercial penaeid species in inland aquaculture at low salinity, extensive research has been conducted on its osmoregulation capabilities. However, inconsistent results have been found in the literature regarding the iso-osmotic point for growth and survival [8–11], [12], immune ability [13], and stress resistance [12, 14].

To understand the molecular mechanism underlying salinity adaptation in *L*. *vannamei*, various genes have been cloned, including those from the gill for ion transport [15], sarco/endoplasmic reticulum Ca^2+^-ATPase [16], glutamate dehydratase [17,18], hyperglycemic hormone [19,20] and molt-inhibiting hormone [20]. Moreover, suppression-subtractive hybridization has been used to identify genes and pathways in juvenile *L*. *vannamei* that have been exposed to long-term low salinity. However, the most common genes in these libraries are immune-related proteins and enzymes [21], whereas genes or pathways related to energy metabolism have not been found.

Whole-transcriptome shotgun sequencing, which is known as RNA sequencing (RNA-seq) [22], has been employed to reveal a snapshot of the transcriptome [23] that can be used to capture and annotate the transcriptome [24], analyze digital gene expression in hemocytes to gain knowledge on the immune response [25] and discover novel transcribed regions in the genomes of aquatic animals [24,26,27]. To understand the complex molecular mechanism of a specific physiological process, RNA-seq is a practical and efficient method of determining nearly all of the genes and pathways involved in a corresponding physiological function [28–30]. In this study, we aimed to compare the transcriptomic response of *L*. *vannamei* to low-salinity stress and reveal the pathways and genes involved in the process of salinity adaption. The results of this study will provide a foundation for understanding the mechanism of osmoregulation in euryhaline crustacean species.

## Results

### Sequencing, *de novo* assembly and differential expression genes

We obtained 97.1 million reads and 98.1 billion nucleotides from the shrimp at both 3 and 30 practical salinity units (psu) (Table 1). After quality trimming and adapter clipping, 93.8 million reads accounting for 96.6% of the total reads were obtained. In addition, 26,034 genes and 38,237 unigenes with an average length of 1,610 bp were obtained by *de novo* assembly using Trinity software after splicing and removing redundancy. Among 26034 genes, 855 genes were significantly up or down regulated, and all the differential expression genes were show in S1 Table. A total of 2,341 unigenes were down-regulated, and 2,363 unigenes were up-regulated in low salinity with the absolute fold change >2. Among the unigenes, the largest and smallest unigenes were 24,554 bp and 351 bp, respectively (Table 2); the length distribution of unigenes is shown in S1 Fig. To assess the quality and coverage of the transcriptome data, we mapped the assembly unigenes by using Bowtie software, and the mapping data accounted for over 90% of the data, suggesting that the transcriptome dataset provided good gene coverage and enriched the transcriptome information of *L*. *vannamei* in the present study.

**Table 1:** Summary of Illumina-expressed short read production and filtered transcriptomic responses to salinity stress in *Litopenaeus vannamei*.

Salinity	Reads	Nucleotides	Q20 (%)	Q30 (%)
3 psu	48,338,820	4,882,220,820	96.50	91.18
30 psu	48,767,738	4,925,541,538	96.68	91.51
Trimmed				
3 psu	46,640,504	4,599,887,662	99.07	94.49
30 psu	47,118,402	4,653,045,098	99.09	94.64

Note: Q20 indicates that every 100 bp of sequencing reads will have an error, and Q30 indicates that every 1000 bp of sequencing reads will have an error.

**Table 2:** Summary of *de novo* assembly results of the transcriptomic responses to salinity stress in *Litopenaeus vannamei*.

Type	Number
Total genes	26034
Total unigenes	38237
Total residues	61573030 bp
Average length	1610.3 bp
Largest unigene	24554 bp
Smallest unigene	351 bp

### Annotation of unigenes

The predicted sequences (predicted open reading frame nucleotide sequences) and unpredictable sequences (unpredictable nucleotide sequences) were annotated using BLASTp and BLASTx, respectively, and then blasted to protein databases, including the nonredundancy (NR), STRING, COG and KOG databases (BLAST 2.2.28+, *E*-value < 1*e*—5) (Table 3). Among the annotated predicted sequences, a total of 17,232 (76.83%), 6298 (28.08%), 3720 (16.58%) and 302 (1.35%) sequences were unambiguous alignments relative to the reference when BLASTx was used against the NR, STRING, KOG, and COG databases, respectively. However, among the unpredictable sequences, only 2,235 (14.14%), 746 (4.72%), 509 (3.22%), 313 (1.98%), 243 (1.54%), 128 (0.81%), and 56 (0.35%) of the 15,806 sequences were matched against the NR, gene ontology (GO), NT, STRING, KOG and COG databases, respectively.

**Table 3:** Summary of the annotations of *Litopenaeus vannamei* unigenes.

	Predicted sequences		Unpredictable sequences	
	Number	Ratio (%)	Number	Ratio (%)
All genes	22431	100	15806	100
Annotated in NR	17232	76.82	2235	14.14
Annotated in NT	None	None	509	3.22
Annotated in GO	None	None	746	4.72
Annotated in string	6298	28.08	313	1.98
Annotated in COG	3720	16.58	128	0.81
Annotated in KOG	5408	24.11	243	1.54
Annotated in NOG	302	1.35	56	0.35

After parsing the GO annotation output, a total of 8,779 unigenes were finally annotated with GO terms, with 50.02% annotated to biological processes, 28.93% annotated to cellular components and 21.05% annotated to molecular functions. The distribution of GO terms showed that cellular processes and metabolic processes were the well-represented terms among the biological processes. Cells and cell parts were significantly enriched in cellular components, and analytic activity and binding consisted of a large proportion of molecular functions (S2 Fig).

Clusters of orthologous groups (COGs) of proteins were determined to predict and classify the possible functions of unigenes. The COG annotation analysis showed that three types of function were obtained, including information storage and processing, cellular processes and signaling, and metabolic pathways. The hits from the COG analysis were functionally classified into 25 categories, and the most enriched terms were related to general functions and then transcription and signal transduction mechanisms (S3 Fig).

### KEGG pathway analysis annotation and functional enrichment analysis of GO and KEGG pathways

**Fig 1:**
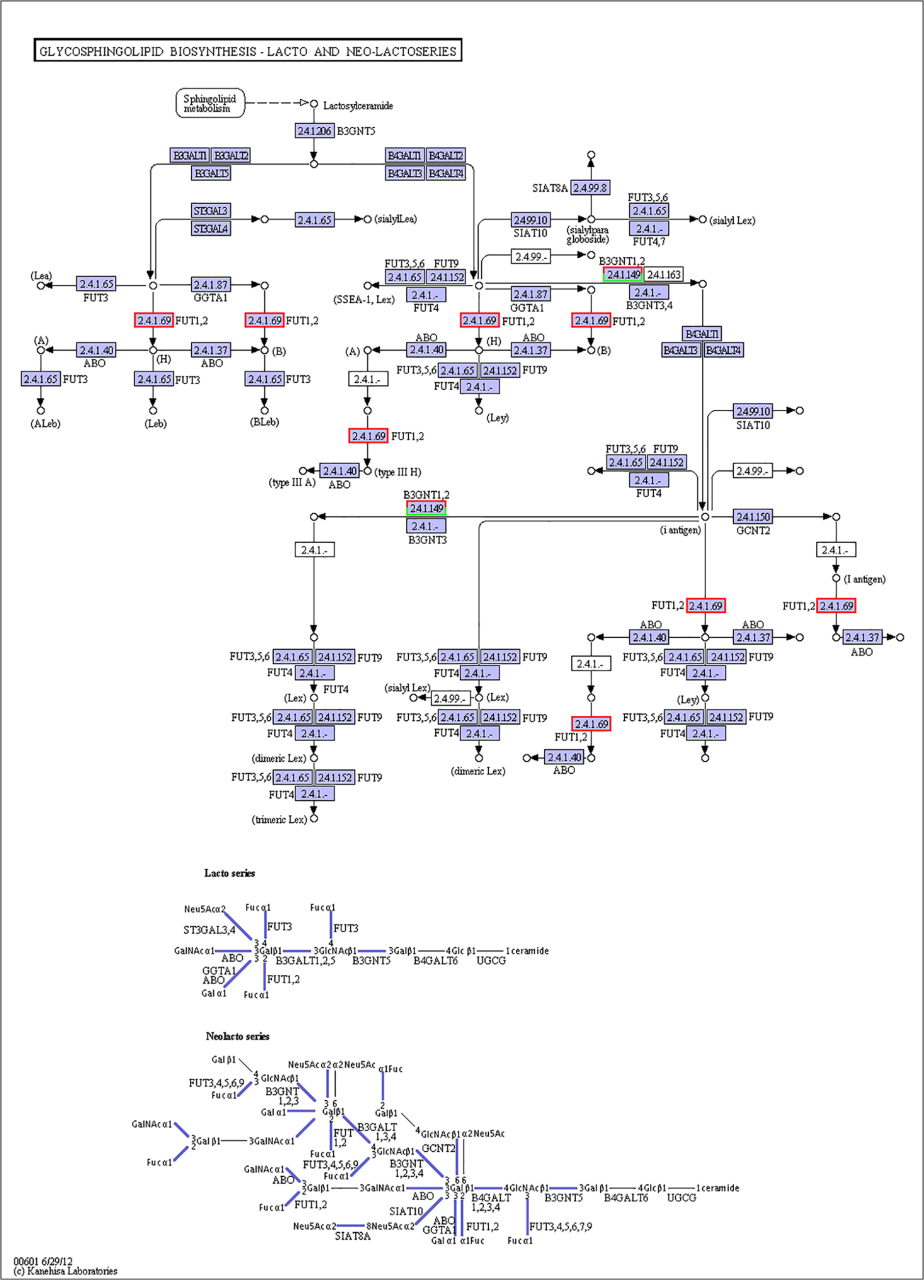
Pathways of glycosphingolipid biosynthesis: lacto and neolacto series (ko00601). The red frames represent the genes were up-regulated, while the green frames represent that the genes were down-regulated. The frames with both red and green indicate that these genes have more than one unigenes, and some of them were up-regulated, but others were down-regulated.

**Fig 2:**
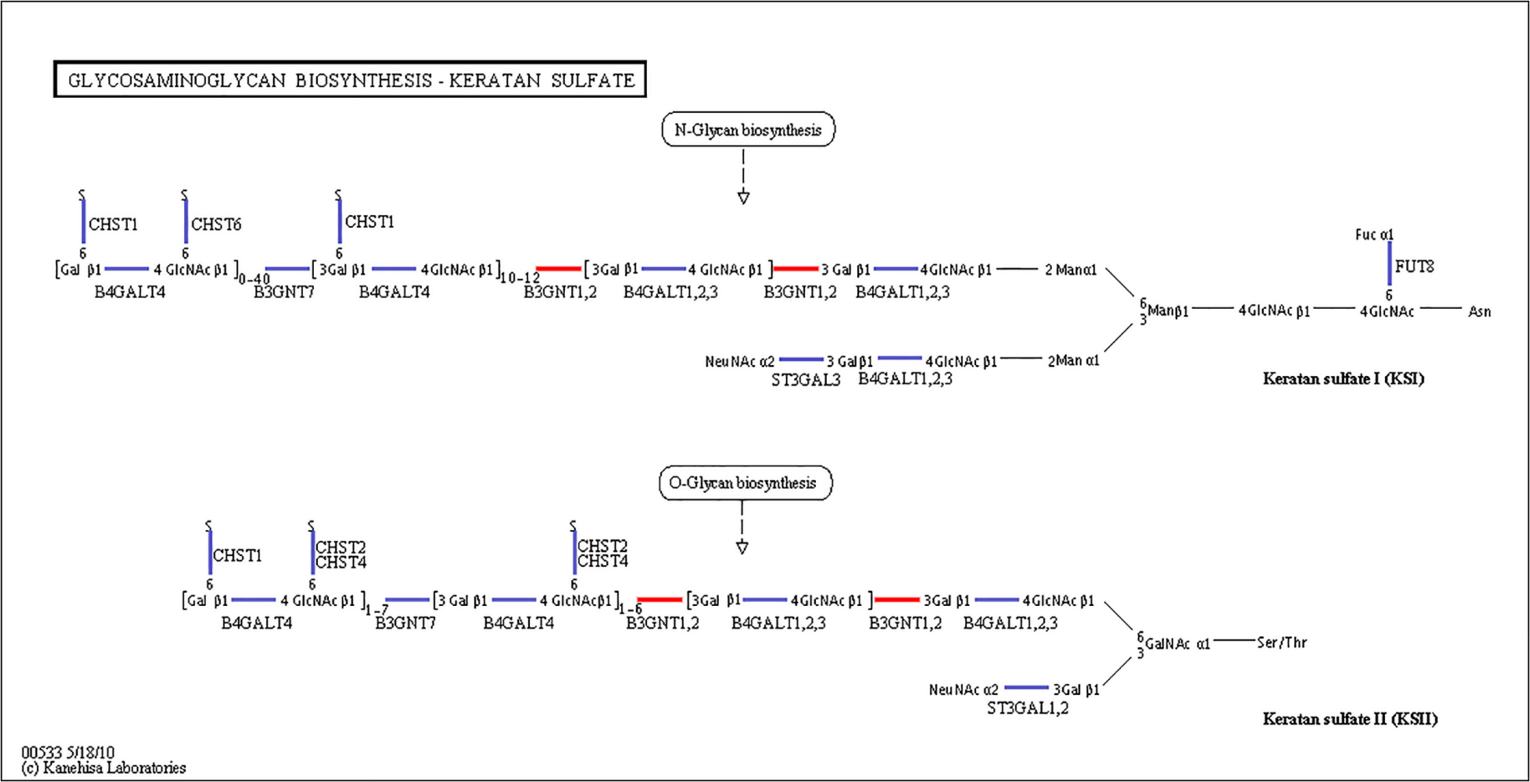
Pathways of glycosaminoglycan biosynthesis: keratan sulfate (ko00533). The red line indicates that the genes were up-regulated.

**Fig 3:**
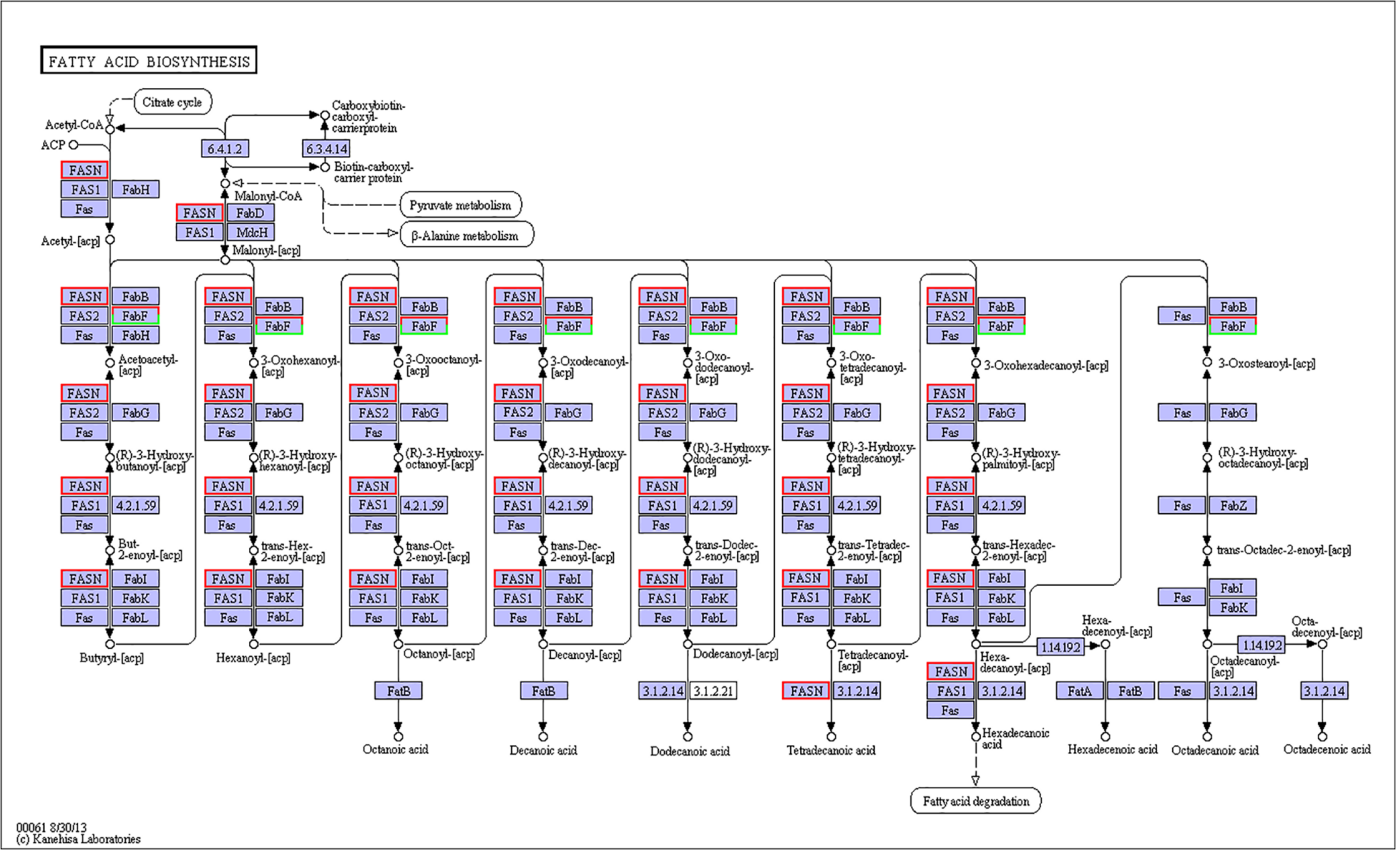
Pathways of fatty-acid biosynthesis (ko00061). The red frames represent the genes were up-regulated, while the green frames represent that the genes were down-regulated. The frames with both red and green indicate that these genes have more than one unigenes, and some of them were up-regulated, but others were down-regulated.

**Fig 4:**
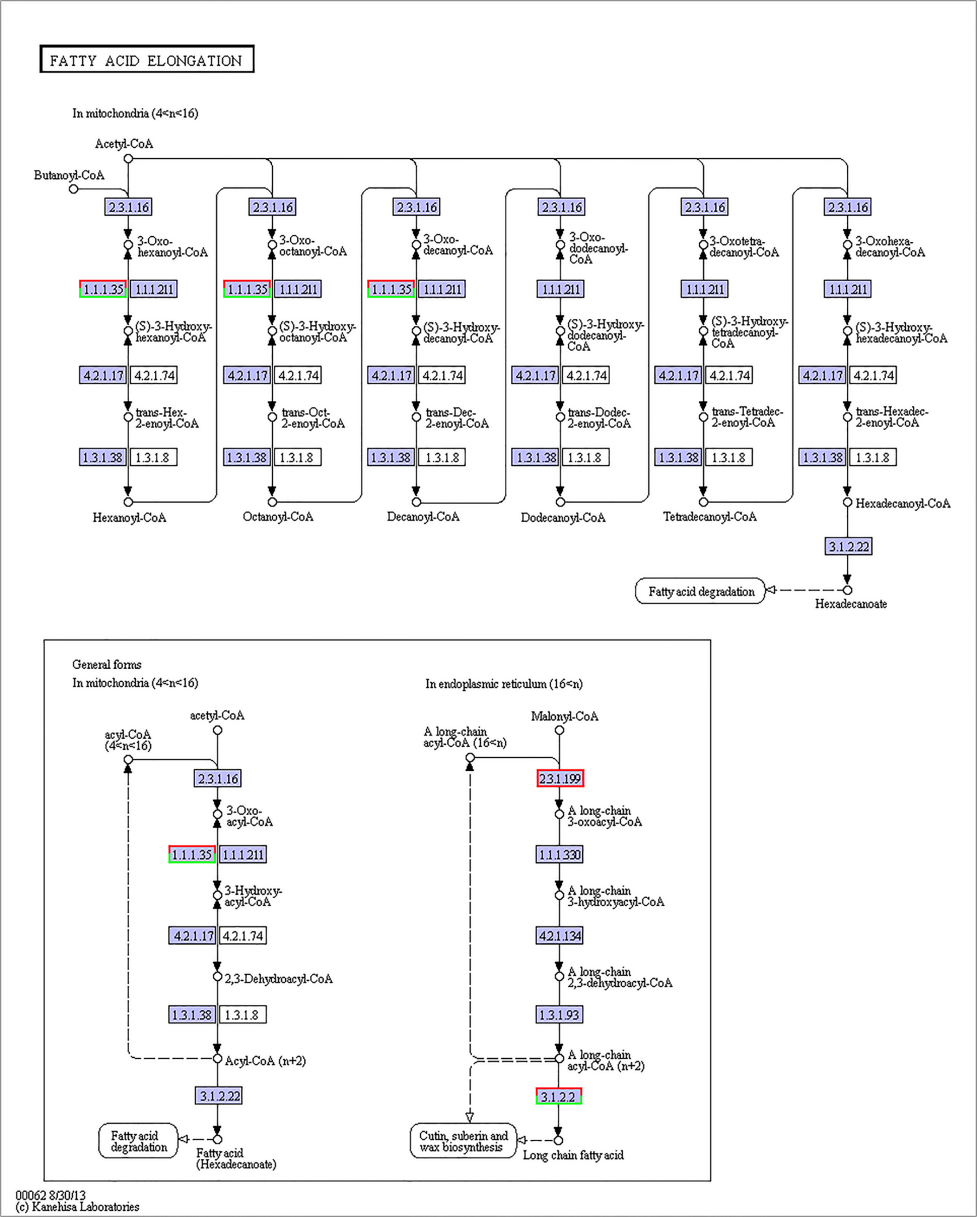
Pathways of fatty-acid elongation (ko00062). The red frames represent the genes were up-regulated, while the green frames represent that the genes were down-regulated. The frames had both red and green indicated that these genes had more than one unigenes, and some of them were up-regulated, others were down-regulated.

**Fig 5:**
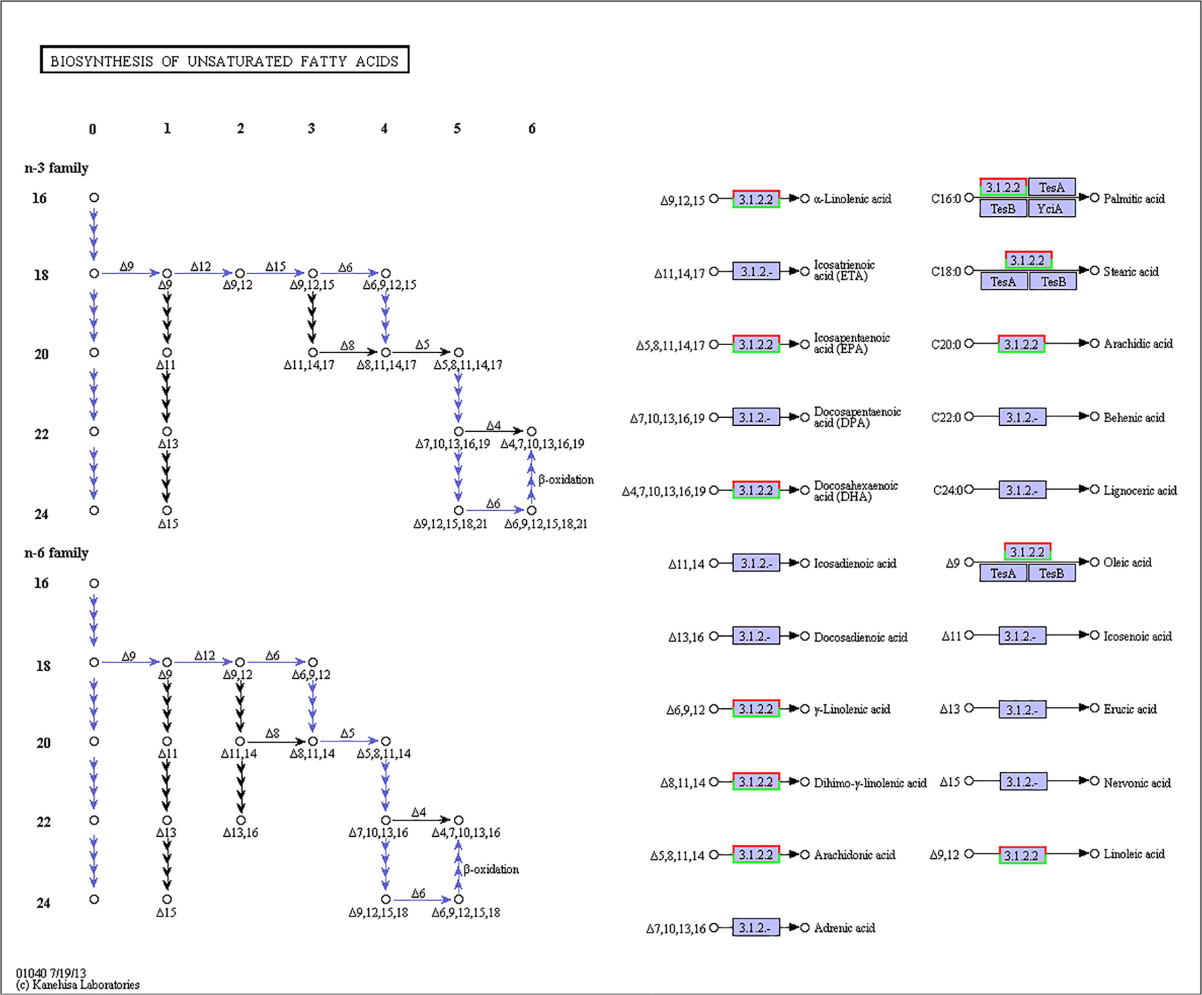
Pathways of unsaturated fatty acid biosynthesis (ko01040). The red frames represent the genes were up-regulated, while the green frames represent that the genes were down-regulated. The frames with both red and green indicate that these genes had more than one unigenes, and some of them were up-regulated, but others were down-regulated.

Various molecular pathways were obtained by Kyoto Encyclopedia of Genes and Genomes (KEGG) annotation. A total of 9,621 unigenes were mapped onto 317 pathways, and the most enriched sequences were metabolic pathways, which was followed by the biosynthesis of secondary metabolites, spliceosome and RNA transport. The top 20 pathways with the greatest number of annotated sequences are shown in Table 4. A total of 47 significantly changed GO terms were obtained, and the most significant change was in molecular functions, which was followed by catalytic activity, histone H4 acetylation, structural constituents of cuticle and chitin binding. All of the significantly changed (*P* < 0.05) GO pathways are listed in Table 5. A KEGG pathway enrichment analysis was performed for the gene expression between salinity treatments to identify the number of significantly changed samples along the pathway that were relevant to the background number. The most significantly changed KEGG pathways were the glycosphingolipid biosynthesis, lysine degradation, glycosaminoglycan biosynthesis and malaria pathways. The gene recorded as B3GNT1,2 was significantly up-regulated both in the glycosphingolipid biosynthesis pathway and glycosaminoglycan biosynthesis pathway when shrimp were exposed to salinity at 3 psu (Figs 1 and 2). In addition, fatty-acid biosynthesis (Fig 3) was significantly enhanced, especially in short-carbon-chain fatty acids (C8-C18). In addition, low-salinity conditions enhanced polyunsaturated fatty-acid (PUFA) biosynthesis, especially that of highly unsaturated fatty acids such as ARA, EPA and DHA (Figs 4 and 5). All of the significantly changed (*P* < 0.05) KEGG pathways are listed in Table 6. These annotations provide valuable information for studying the specific biological and metabolic processes and functions and molecular mechanisms under salinity stress in *L*. *vannamei*.

**Table 4:** The top 20 pathways with the greatest number of annotated sequences.

Pathway ID	Pathway definition	Number of sequences
path:ko01100	Metabolic pathways	1476
path:ko01110	Biosynthesis of secondary metabolites	419
path:ko03040	Spliceosome	278
path:ko03013	RNA transport	260
path:ko04144	Endocytosis	247
path:ko05169	Epstein-Barr virus infection	245
path:ko00230	Purine metabolism	236
path:ko05205	Proteoglycans in cancer	232
path:ko05200	Pathways in cancer	231
path:ko01120	Microbial metabolism in diverse environments	227
path:ko04142	Lysosome	219
path:ko05166	HTLV-I infection	216
path:ko04151	PI3K-Akt signaling pathway	216
path:ko04510	Focal adhesion	215
path:ko04141	Protein processing in endoplasmic reticulum	187
path:ko05016	Huntington's disease	186
path:ko04530	Tight junction	185
path:ko05203	Viral carcinogenesis	180
path:ko05168	Herpes simplex infection	180
path:ko04120	Ubiquitin mediated proteolysis	172

**Table 5:** Significantly changed GO pathways of *L*. *vannamei* between the two salinities.

GO ID	Pathway description	*P* value	Type
GO:0003674	molecular function	1.88E-06	Molecular function
GO:0003824	catalytic activity	2.12E-06	Molecular function
GO:0043967	histone H4 acetylation	0.000136	Biological process
GO:0042302	structural constituent of cuticle	0.000223	Molecular function
GO:0008061	chitin binding	0.000638	Molecular function
GO:0097367	carbohydrate derivative binding	0.000884	Molecular function
GO:0006030	chitin metabolic process	0.0016	Biological process
GO:1901071	glucosamine-containing compound metabolic process	0.0021	Biological process
GO:0006022	aminoglycan metabolic process	0.0041	Biological process
GO:0016573	histone acetylation	0.0047	Biological process
GO:0018393	internal peptidyl-lysine acetylation	0.0047	Biological process
GO:0018394	peptidyl-lysine acetylation	0.0047	Biological process
GO:0006475	internal protein amino acid acetylation	0.0061	Biological process
GO:0000123	histone acetyltransferase complex	0.0075	Cellular component
GO:0006473	protein acetylation	0.0079	Biological process
GO:0043543	protein acylation	0.0129	Biological process
GO:1901564	organonitrogen compound metabolic process	0.0139	Biological process
GO:0018205	peptidyl-lysine modification	0.0139	Biological process
GO:0006040	amino sugar metabolic process	0.0163	Biological process
GO:0008152	metabolic process	0.0188	Biological process
GO:0016491	oxidoreductase activity	0.0314	Molecular function
GO:0005576	extracellular region	0.0476	Cellular component

**Table 6:** Significantly changed KEGG pathways of *L*. *vannamei* between the two tested salinities.

Pathway description	KEGG ID	Sample number	Background number	P-value
Glycosphingolipid biosynthesis—lacto and neolacto series	ko00601	7	16	0.001
Lysine degradation	ko00310	23	102	0.002
Glycosaminoglycan biosynthesis—keratan sulfate	ko00533	7	17	0.002
Malaria	ko05144	11	41	0.007
Phosphonate and phosphinate metabolism	ko00440	4	8	0.009
Glycerophospholipid metabolism	ko00564	20	98	0.011
Steroid hormone biosynthesis	ko00140	8	29	0.017
Glycosaminoglycan degradation	ko00531	11	48	0.023
Adipocytokine signaling pathway	ko04920	14	67	0.025
Fatty-acid biosynthesis	ko00061	3	6	0.026
Synthesis and degradation of ketone bodies	ko00072	8	32	0.031
Ether lipid metabolism	ko00565	9	38	0.031
Drug metabolism—cytochrome P450	ko00982	13	63	0.033
Drug metabolism—other enzymes	ko00983	15	76	0.033
TGF-beta signaling pathway	ko04350	12	57	0.034
Arachidonic acid metabolism	ko00590	14	70	0.035
Metabolism of xenobiotics by cytochrome P450	ko00980	14	71	0.039
Cholinergic synapse	ko04725	14	71	0.039

Note: The sample number means differently expressed gene number, and the background number means the total gene number in the pathway.

### Validation of RNA-seq profile results by qPCR

**Fig 6:**
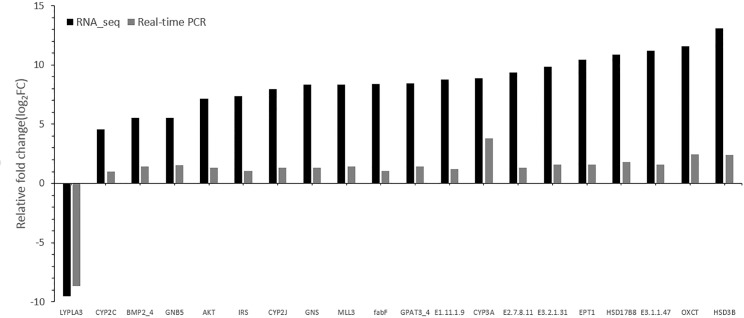
Validation results of RNA-seq profiles by qPCR.

Twenty randomly selected genes were measured in the same hepatopancreas RNA samples by qPCR, and the expression levels of these genes were significantly associated with the RNA-seq results (R = 0.91, Fig 6). These results confirm the reliability of RNA-seq and accuracy of the Trinity assembly.

## Discussion

Osmoregulation in crustaceans is a complex process because of the diverse range of salinities that they are exposed to in their natural habitat. High-throughput RNA-seq is a good method in determining the underlying molecular mechanisms of osmoregulation [31]. In addition, transcriptome analysis has been used in the discovery of single nucleotide polymorphisms (SNPs) [32] and identification of immune gene [33] and genes and pathways responsible for various pathogen challenges [34–37] in *L*. *vannamei*. In the present study, we used RNA-seq on *L*. *vannamei* exposed to low salinity to determine the metabolic pathways and perform expression profiling. We found that most of the metabolic activities that were significantly involved in adaptive responses were related to lipid metabolism. This study is the first of its type to report on the key energetic pathways relevant to osmoregulation in *L*. *vannamei*.

### Relationship between lipid metabolism and response strategies to salinity stress

Shrimp gill is one of the main tissues for osmoregulation and icon exchange, and the polyunsaturated fatty acids synthesized in hepatopancreas are the main components of shrimp gill. Previous study has shown that under salinity stress, shrimp gill needs extra energy (most from saturated fatty acids) and polyunsaturated fatty acids to ensure the osmoregulation and icon exchange in *L*. *vannamei* [38]. In this study, a number of lipid metabolism pathways in the hepatopancreas of *L*. *vannamei* were influenced by salinity stress, including the fatty-acid biosynthesis pathway, arachidonic acid metabolism pathway (S4 Fig), adipocytokine signaling pathway (S5 Fig), glycerophospholipid metabolism pathway (S6 Fig), ether lipid metabolism pathway (S7 Fig) and ketone body synthesis and degradation pathway (S8 Fig). These pathways are found in the Chinese mitten crab *Eriocheir sinensis* under osmotic stress [31]. Lipids, including the fatty-acid structure, composition and metabolism, reduce osmotic shock in aquatic animals (Sui et al., 2007) by providing sufficient energy to maintain the ion balance and regulate the structure of biological membranes [6,39]. This process can directly or indirectly function in “compensatory processes” and “limiting processes," which are the two major osmoregulation strategies in crustaceans [4, 40].

In this study, most of the significant pathways related to salinity adaptations were associated with the above two major strategies. The limiting process is a strategy to adjust the permeability of the boundary structures to maintain hemolymph osmolality/ions in gill membranes, which is effective at reducing ion diffusion and water influx because the ion transport mechanism requires additional energy [4]. The ability to regulate gill permeability is an adaptive response that is crucial for decapod crustaceans because it facilitates long-term habitation in environments with variable salinity [4, 40]. Therefore, the cell membranes of the gill should play an important role in osmoregulation [41]. The “compensatory process” strategy is accomplished via the active exchange of solutes in hemolymph to counterbalance passive diffusion and maintain osmolality [4]. Because this process is energetically costly, a number of energy metabolism pathways and ion regulation pathways must be involved.

### Energy from lipid metabolism to maintain the ion balance

Fatty-acid biosynthesis was significantly enhanced, especially for short-carbon-chain fatty acids (C8-C18), and arachidonic acid was also used to convert fatty acids or other metabolic products in the present study. When shrimp suffer from ambient salinity stress, additional energy is required through nutrient intake to maintain homeostasis by osmoregulation via the “compensatory process,” in which lipids play significant roles [42–45].

However, ketone bodies are indispensable for energy that is metabolized from fatty acids, which was revealed by the KEGG analysis in this study. Ketone bodies are produced by the liver from fatty acids during periods of low food intake or carbohydrate restriction. When carbohydrates are scarce, energy must be obtained from the breakdown of fatty acids from body tissue instead of glucose [46, 47]. Interestingly, in the adipocytokine signaling pathway, we found that the gene FACS functioned in long-chain fatty-acid biosynthesis and was significantly down-regulated under low-salinity stress. CPT-1 is another relevant gene for long-chain fatty-acid *β*-oxidation, and it was significantly down-regulated. Therefore, it is reasonable to assume that *L*. *vannamei* prefers to use shorter-chain fatty acids for energy supplementation and selectively retains longer-chain unsaturated fatty acids [39, 45]. The enhancement of saturated fatty acid biosynthesis in hepatopancreas (the main site for lipogenesis) would provide sufficient energy for osmoregulation in shrimp gill at low salinity, and this result is consistent with our previous findings in *L*. *vannamei* cultured at low salinity [38]. *Penaeus monodon* also prefers to use shorter-chain fatty acids in energy metabolism and selectively retains longer-chain unsaturated fatty acids [48].

### Polyunsaturated fatty acids in osmoregulation of *L*. *vannamei*

In this study, fatty acid elongation (n > 18) was significantly enhanced, and the biosynthesis of α-linolenic acid, EPA, DHA, arachidic acid, arachidonic acid and other PUFAs was up-regulated at low salinity. Saturated fatty-acid biosynthesis was enhanced, and PUFA biosynthesis was also strengthened at low salinity. PUFAs can improve the resistance to osmotic shock in aquatic animals because PUFAs are mainly incorporated in cell membranes and can increase membrane permeability and fluidity [49, 50]. Free fatty acids, especially LC-PUFA, have the potential to modulate fatty-acid composition in the gill membrane and increase enzymatic efficiency [45, 51]. Modifications to the fatty-acid composition in the gills that increase the level of LC-PUFA can increase the gill area to enhance the osmoregulatory capacity in shrimp at low salinity, thereby increasing survival [39]. In addition, arachidonic acids in fish can enhance the branchial Na^+^/K^+^-ATPase activity and influence the ion balance [52]. Furthermore, arachidonic acid metabolites can influence the regulation of ion balances across the gill membrane [53].

### Glycosphingolipid and glycosaminoglycan metabolism pathways in regulating membrane structure

In this study, the most influential pathways were associated with glycosphingolipid biosynthesis, glycosaminoglycan biosynthesis and glycosaminoglycan degradation, and they are all related to gill permeability. Glycosphingolipids (including lacto-/neolactoglycolipids and sphingolipids) function to protect the cell surface by maintaining the stability of the membrane or plasma membrane to protect against harmful environmental factors by forming a mechanically stable and chemically resistant outer leaflet for the plasma membrane lipid bilayer [54, 55]. Glycosaminoglycans are classified into four groups: keratan sulfate, heparin/heparan sulfate, chondroitin/dermatan sulfate and hyaluronic acid. One of the main functions of keratan sulfates is the maintenance of tissue hydration and implantation and migration of endothelial cell [56].

When shrimp were exposed to salinity of 3 psu, the B3GNT1,2 gene, which participates in both the glycosphingolipid biosynthesis pathway and glycosaminoglycan biosynthesis pathway, was significantly up-regulated. The FUT1_2 gene was also significantly up-regulated in the glycosphingolipid biosynthesis pathway. Thus, we believe that the genes B3GNT1,2 and FUT1_2 can promote glycolipid biosynthesis (Fig 1). Glycolipids constitute the lipid bilayer of the plasma membrane and play an important role in maintaining the physical state of the membrane [57]. Thus, glycolipid biosynthesis might promote the “limiting process” and provide resistance to low-salinity stress at 3 psu salinity. However, the effect of keratan sulfate and glycosaminoglycan degradation in low-salinity ambient osmoregulation is still not clear and requires further study.

Glycerophospholipids are glycerol-based phospholipids that are the main component of biological membranes [58], and their biosynthesis was significantly enhanced at low salinity (Fig 1). Although direct interactions were not observed between glycerophospholipid and osmoregulation in this study, glycerophospholipids could indirectly improve osmoregulation by affecting membrane permeability. Ether lipids are ubiquitous and constitute a major portion of the cell membranes in mammals [59]. In addition, these lipids play an important role in the generation of lipid second messenger systems, such as prostaglandins and arachidonic acid, which are important in signal transduction [60]. Ether lipids also act directly in cell signaling [61] and are involved in osmotic stress signal transduction. Another possible function of ether lipids is as an antioxidant against oxidative stress, which has been demonstrated in cell culture under salinity stress. Therefore, these lipids might play a role in serum lipoprotein metabolism [62] and lipid metabolism may play an important and indispensable role in osmoregulation [38].

### Potential pathways in *L*. *vannamei* under chronic low salinity stress

Because of the complexity of the physiological response to low-salinity stress in *L*. *vannamei*, several pathways (in addition to lipid metabolism) show potential importance in the shrimp's ability to cope with salinity stress. However, clear evidence of the direct involvement of these pathways during salinity challenges has not been observed; thus, the putative functions of these pathways including lysine degradation, cholinergic synapse, drug metabolism pathway, steroid hormones metabolism pathway, phosphonate and phosphinate metabolism, are only briefly discussed.

Lysine is metabolized in eukaryotes to yield acetyl-CoA via lysine acetylation [63, 64]. Acetyl-CoA participates in osmoregulation as an intermediate metabolite commonly produced from energy metabolism, lipid metabolism and carbohydrate metabolism. In our results, the gene expression of 3-hydroxyacyl-CoA dehydrogenase, which can produce acetyl-CoA, was significantly up-regulated, resulting in more acetyl-CoA entering the citrate cycle for energy production. The above pathway could indirectly influence ion transfer or energy metabolism and promote “compensatory processes," which is consistent with reports on the Chinese mitten crab under salinity stress [31]. On the other hand, the choline in animal tissues is a primary component of neurotransmitter acetylcholine and functions with inositol as a basic constituent of lecithin [65]. Choline prevents the formation of fat deposits in the liver and facilitates the movement of fat into cells [66]. Thus, considering the result of our previous study [38], it seems that more fat may be used in supplying energy through β-oxidation.

Various physiological and pathological factors can affect drug metabolism, including age, individual variations, enterohepatic circulation, nutrition, intestinal flora and sex differences. Cytochrome P450 influences arachidonic acid metabolism [67, 68] and fatty-acid metabolism [69] and may have an indirect influence on osmoregulation by influencing the regulation of fatty-acid metabolism and other physiological and biochemical processes. However, the effect of drug metabolism on osmoregulation is still unknown and requires further study. Among the malaria pathways, we found that low-density lipoprotein receptor-related protein 1 (LRP1) was significantly changed when shrimp suffered from salinity stress. LRP1 might have an important role in low-density lipoproteins and thereby influence osmoregulation.

Steroid hormones help control metabolism, inflammation, immune functions and salt and water balance, influence sex characteristics and promote illness and injury prevention [70–72]. Moreover, Birukawa found that steroid hormones are involved in the osmoregulation of cetaceans [73]. Phosphonates are effective chelating agents that remain stable under harsh conditions. Phosphonates are also regularly used in reverse-osmosis systems [74]. However, the interaction or correlation between osmoregulation and phosphonates is still not clear and requires further study in aquatic animals.

## Conclusion

**Fig 7:**
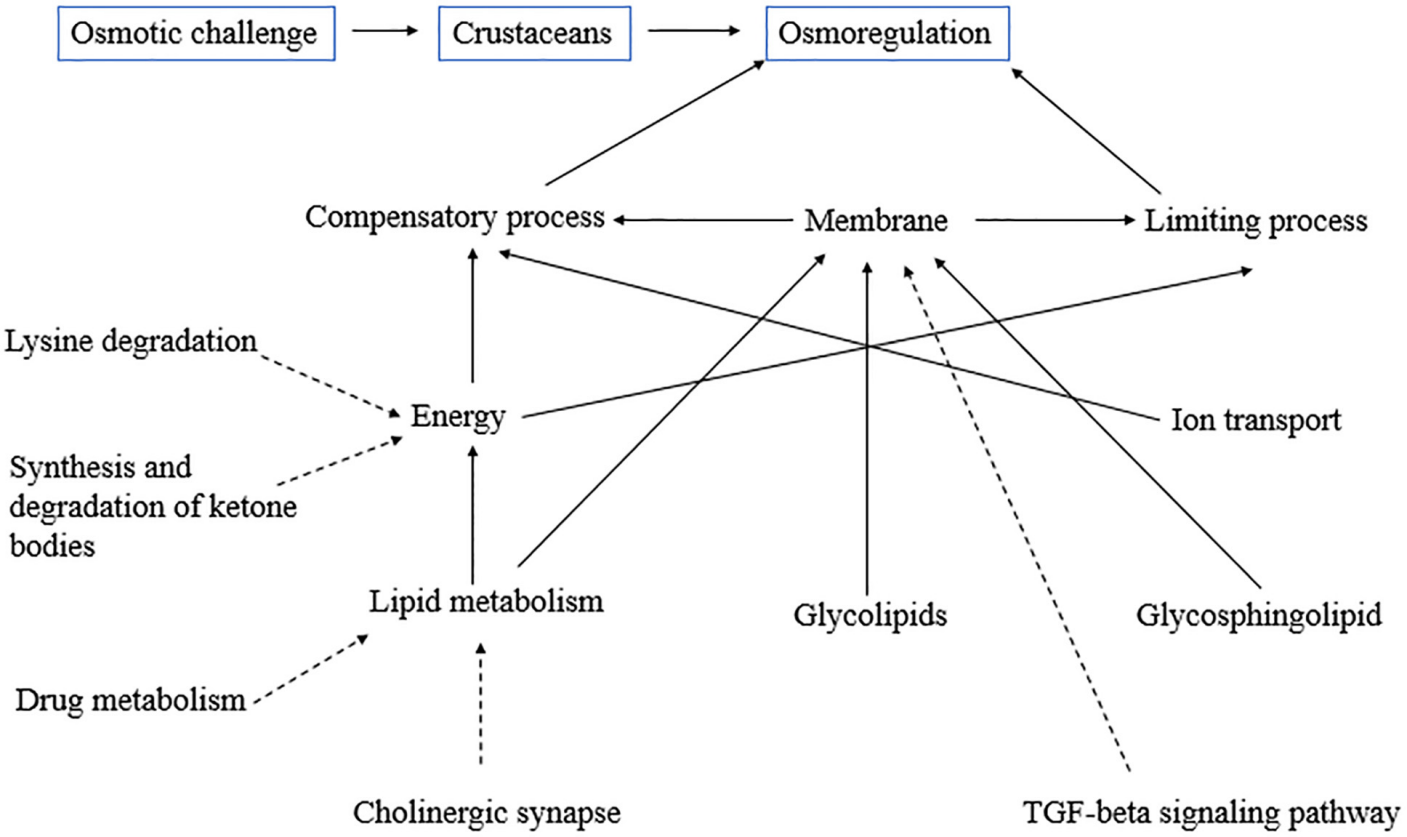
Relationship between the most influenced pathways and osmoregulation. The dotted-line arrows are indirect effects, and solid-line arrows indicate direct influence.

This study revealed that osmoregulation is a complex physiological adaptation that involves a number of pathways, especially the lipid metabolism pathway. When *L*. *vannamei* is subjected to an osmotic challenge at low salinity, shrimp can improve itself ability against osmotic stress by both “compensatory processes” and “limiting processes," which are the two major osmoregulatory strategies in crustaceans. These two osmoregulatory strategies may utilize various osmoregulatory mechanisms that are largely dependent on energy metabolism and cell membrane regulation (Fig 7). Not only will lipid metabolism supply sufficient energy to ensure the energy metabolism demand, but also many pathways (e.g. lysine degradation, cholinergic synapse, drug metabolism etc.) may indirect affect energy metabolism by generating some metabolic intermediates to indirectly influence the energy metabolism or affect lipid metabolism. On the other hand, other lipid metabolism pathways, including glycolipids and glycosphingolipid, are involved to improve osmoregulation capacity by increasing related enzymatic activity or changing gill permeability. This study provides some insights into the pathways involved in *L*. *vannamei* osmoregulation under low salinity. However, the mechanisms underlying osmoregulation in *L*. *vannamei* are complex and require further study, especially in relation to lipid metabolism and osmoregulation.

## Materials and Methods

### Experimental animals, design and facilities

Juvenile white shrimp (1.98 ± 0.28 g) were obtained from the Shenzhen base of the South China Sea Fisheries Research Institute, Shenzhen, China, and they were stocked in six tanks at a density of 40 shrimp per tank (500 L) at 17 psu salinity for one week. The shrimp were acclimated to 3 psu and 30 psu in three tanks through daily increments of 2 psu prior to the start of the 8-week experiment. During the acclimation and experimental periods, the shrimp were fed a commercial diet (10% moisture, 40% crude protein, 8% crude lipid, 12% ash, 30% carbohydrates, 16.7 kJ g^-1^ digestible energy) three times daily at 08:00, 16:00 and 22:00 h. Based on the amount of feed left over from the previous day, daily rations were adjusted to a feeding level slightly over satiation. The unused feed was removed daily with a siphon tube. The photoperiod was 12 h light and 12 h dark. Seawater was pumped from the Dayawan Coast in Shenzhen and filtered through an activated carbon cartridge for at least 3 d before entering the culture system. The tap water was aerated before it was added to the tank to adjust the salinity level. During the experiment, water equal to 1/3 of the tank volume was exchanged once daily. The water quality parameters were monitored 2–3 times a week throughout the feeding trial and maintained at pH 7.5–7.9, temperature 26–28°C, dissolved oxygen 4.8–6.4 mg L^-1^, and total ammonia nitrogen < 0.02 mg L^-1^ during the trial.

At the end of the experiment, shrimp were fasted for 24 h before sampling. Five shrimp at the intermolt stage C in each tank were dissected to obtain the hepatopancreas tissue for the transcriptome analysis.

### Eukaryote *de novo* transcriptome sequencing

Total RNA was extracted from the hepatopancreas using the TRIzol reagent (Invitrogen) according to the manufacturer’s instructions, and genomic DNA was removed using DNase I (TaKaRa). RNA quality was determined by a 2100 Bioanalyzer (Agilent), and the RNA was quantified using the NanoDrop 2000 spectrophotometer (ND-2000, Gene Company Ltd.). Only high-quality RNA samples (OD_260/280_ = 1.8–2.2, OD_260/230_ ≥ 2.0, RIN ≥ 6.5, 28S:18S ≥ 1.0, > 10 μg) were used to construct the sequencing library.

### Library preparation and Illumina Hiseq2000 sequencing

The RNA-seq transcriptome library was prepared following the TruSeq RNA (Illumina, San Diego, CA) sample preparation instructions using 5 μg total RNA. Briefly, messenger RNA was isolated according to the polyA selection method by oligo beads [75] and then segmented (100 bp to 400 bp) by a fragmentation buffer. Next, double-stranded cDNA was synthesized using a SuperScript double-stranded cDNA synthesis kit (Invitrogen) with random hexamer primers (Illumina). Then, the synthesized cDNA was subjected to end repair, phosphorylation and ‘A’ base addition according to Illumina’s library construction protocol. The libraries were size-selected for cDNA target fragments of 200–300 bp on 2% low-range ultra-agarose followed by PCR amplification using Phusion DNA polymerase (New England Biolabs) for 15 PCR cycles. After quantification by a TBS-380 fluorometer, the paired-end RNA-seq library was sequenced with the Illumina HiSeq 2000 system (2 × 100 bp read length). Raw reads were archived at the National Center for Biotechnology Information (NCBI) Sequence Read Archive under the accession No. SRP048814.

### *De novo* assembly and annotation

The raw paired-end reads were trimmed and quality controlled by SeqPrep (https://github.com/jstjohn/SeqPrep) and Sickle (https://github.com/najoshi/sickle) with default parameters. Clean data from the samples were then used to perform RNA *de novo* assembly with the program Trinity (http://trinityrnaseq.sourceforge.net/) [76]. All of the assembled transcripts were searched against the NCBI protein NR, STRING and KEGG databases using BLASTx to identify the proteins that had the highest sequence similarity with the given transcripts to retrieve their function annotations, and a typical E-value cut-off was set at < 1.0×10^−5^. BLAST2GO (http://www.blast2 go.com/b2 ghome) [77] was used to obtain the GO annotations of uniquely assembled transcripts for describing biological processes, molecular functions and cellular components. A metabolic pathway analysis was performed using KEGG databases (http://www.genome.jp/kegg/) [78, 79].

### Differential expression analysis and functional enrichment

To identify differentially expressed genes (DEGs) between two different samples, the expression level of each transcript was calculated according to the fragments per kilobase of exon per million mapped reads (FRKM) method. RSEM (http://deweylab.biostat.wisc.edu/rsem/) [80] was used to quantify gene and isoform abundances, and the R statistical package software EdgeR (empirical analysis of digital gene expression in R, http://www.bioconductor.org/packages/2.12/bioc/html/edgeR.html) [81] was used for the differential expression analysis. A functional-enrichment analysis that KEGG was performed to identify the DEGs that were significantly enriched in GO terms and metabolic pathways (at a Bonferroni-corrected P-value 0.05) relative to the whole-transcriptome background. The GO functional enrichment and KEGG pathway analysis were conducted by Goatools (https://github.com/tanghaibao/Goatools) and KOBAS (http://kobas.cbi.pku.edu.cn/home.do) [82].

### Experimental validation of RNA-seq profiles by qPCR

Twenty randomly selected genes with significant expression in KEGG pathways were validated with quantitative real-time PCR (qPCR) and gene-specific primers designed using Primer Premier 6 (Table 7). Total RNA was extracted from the target hepatopancreas tissues using a TRIpure Reagent kit (Aidlab, RN01). Samples of polyadenylated RNA were reverse-transcribed using a TaKaRa kit (Cat. No. RR036A). The reactions were conducted in a total volume of 20 μl with the following reaction components: 2 μl 5X PrimeScript RT Master Mix (Perfect Real Time), 1 μg total RNA, and RNase-free dH_2_O up to 20 μl. The protocol for reverse transcription was 37°C for 15 min and 85°C for 5 s. The qPCR analysis was conducted in the CFX96 Real-Time PCR system (Bio-Rad Laboratories, Richmond, CA) using the Ultra SYBR Mixture (WCBIO, CW0957). The amplifications were performed in a 96-well plate in a 20 μl reaction volume containing 10 μl Ultra SYBR Mixture (WCBIO, CW0957), 0.4 μl each gene-specific forward and reverse primer, 8.4 μl RNase-free water and 0.8 μl cDNA. The thermal profile for the Ultra SYBR Mixture PCR analysis was 95°C for 10 min followed by 40 cycles of 95°C for 15 s and 60°C for 1 min. The β-actin gene was used as the reference, and each gene had three replicated wells. Relative fold changes were calculated in the Relative Expression Software Tool version 2009 based on the cycle threshold values generated by qPCR [83].

**Table 7:** Primers used for the qPCR analysis.

Gene name		Product size	Primer sequences (5’-3’)
MLL3	F	90	GACATCTCCTACCACATATACT
	R		TTGACATACAGCACACCAT
LYPLA3	F	121	TGGAACAGTCAACCTAAGAA
	R		GTCAGAGTCACGCAAGAT
GPAT3_4	F	147	TAGCAAGGAGATTACGAGAG
	R		TGGCGACTGGATAGATGA
E2.7.8.11	F	132	ATTCTCCGCATCTACTACAC
	R		CCTCCAGAGTCCTATTCCA
EPT1	F	117	ATGACCAAGAGCGAGATG
	R		ACAGACACAATACAGGAGAT
HSD3B	F	95	CCAACACAATGCCTTCCT
	R		CTTCCTCAGAGCCATGAC
HSD17B8	F	98	AGAGAAGGAGCACGAGTG
	R		CTTACCGCCAGATGATTATTG
CYP3A	F	139	TAGGCATCATAGGCAGGAA
	R		TCTGGCAGGTTGTCTTCA
fabF	F	120	AGCCATCCTCACCATTCT
	R		TATTCTTCTGTCCGCCATC
E3.1.1.47	F	145	GAGCACAGAGACAGTTCC
	R		CTGGCTGTTCCTGAGTTC
E1.11.1.9	F	119	CTGAATGGCGTCCGTTAC
	R		CGAAGTCTGTGTCTGTGTAT
CYP2J	F	106	TCCTACCAGCACAAGAGT
	R		GCCAGGTAAGTGTCAGTC
IRS	F	114	AGAGGAGAGTGCCATATCA
	R		ACCGCTGTTGTTAGTTGT
AKT	F	118	AGCACGAGACCTCCTTAG
	R		CAGTTGATGGTGATGTAGAAG
GNS	F	149	GAGGACTCGTGGAACAAC
	R		CGCTCTTCAGGTCATACAT
E3.2.1.31	F	129	ATTCGCACTCTTGGATGG
	R		CACTTGAGGAGGCTGAAG
OXCT	F	126	AACGGACGGAATTACATCA
	R		GCACATTGGTAGGTTGAAG
CYP2C	F	125	CTGACGGCTCTGTATCTG
	R		TGTGCTTGATGTGGTCTC
BMP2_4	F	122	ACCAATACCTCGCTGATG
	R		GATGTTCGTCACGTTGAAG
GNB5	F	106	GCAGGATACAACGACTACA
	R		GAGACATCTTCAGACAGGAG

## Supporting Information

S1 Fig The length distribution of isogenes by Illumina sequencing. (TIF)

S2 Fig Gene Ontology distribution of isogenes annotated with GO terms. (TIF)

S3 Fig Distribution of isogenes annotated with COG terms. (TIF)

S4 Fig Pathway of arachidonic acid metabolism (ko00590). (TIF)

S5 Fig Pathway of adipocytokine signaling pathway (ko04920). (TIF)

S6 Fig Pathway of glycerophospholipid metabolism. (TIF)

S7 Fig Pathway of ether lipid metabolism. (TIF)

S8 Fig Pathway of synthesis and degradation of ketone bodies. (TIF)

S1 Table The differential expression genes. (XLS)
