# Supplementary figures and images for: Transcriptome and Molecular Pathway Analysis of the Hepatopancreas in the Pacific White Shrimp Litopenaeus vannamei under Chronic Low-Salinity Stress
Source: PLoS One. 2015 Jul 6;10(7):e0131503. doi: 10.1371/journal.pone.0131503 (PMC4492601; doi:10.1371/journal.pone.0131503)

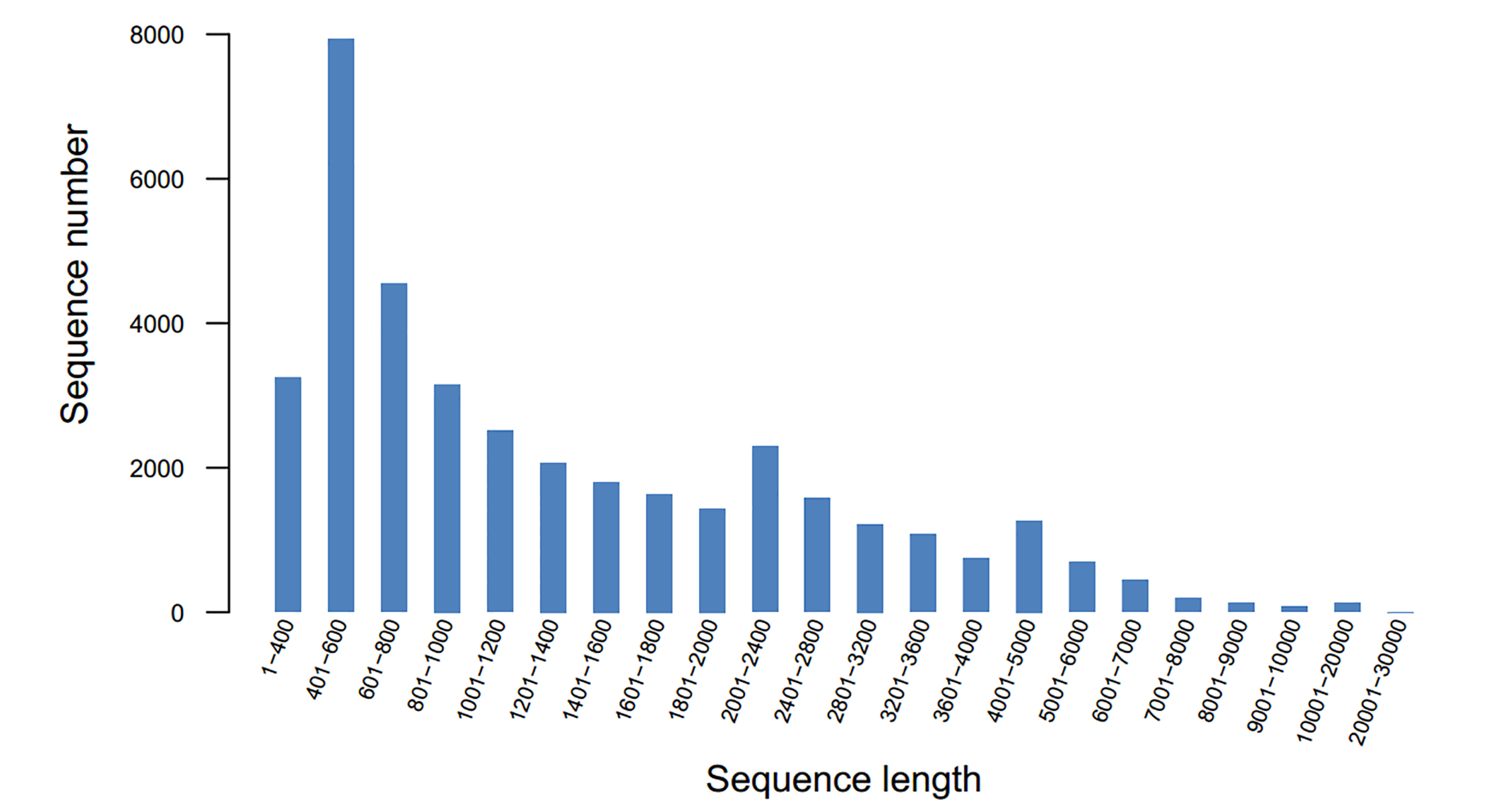

Supplement: S1 Fig — (TIF) [file pone.0131503.s001.tif]

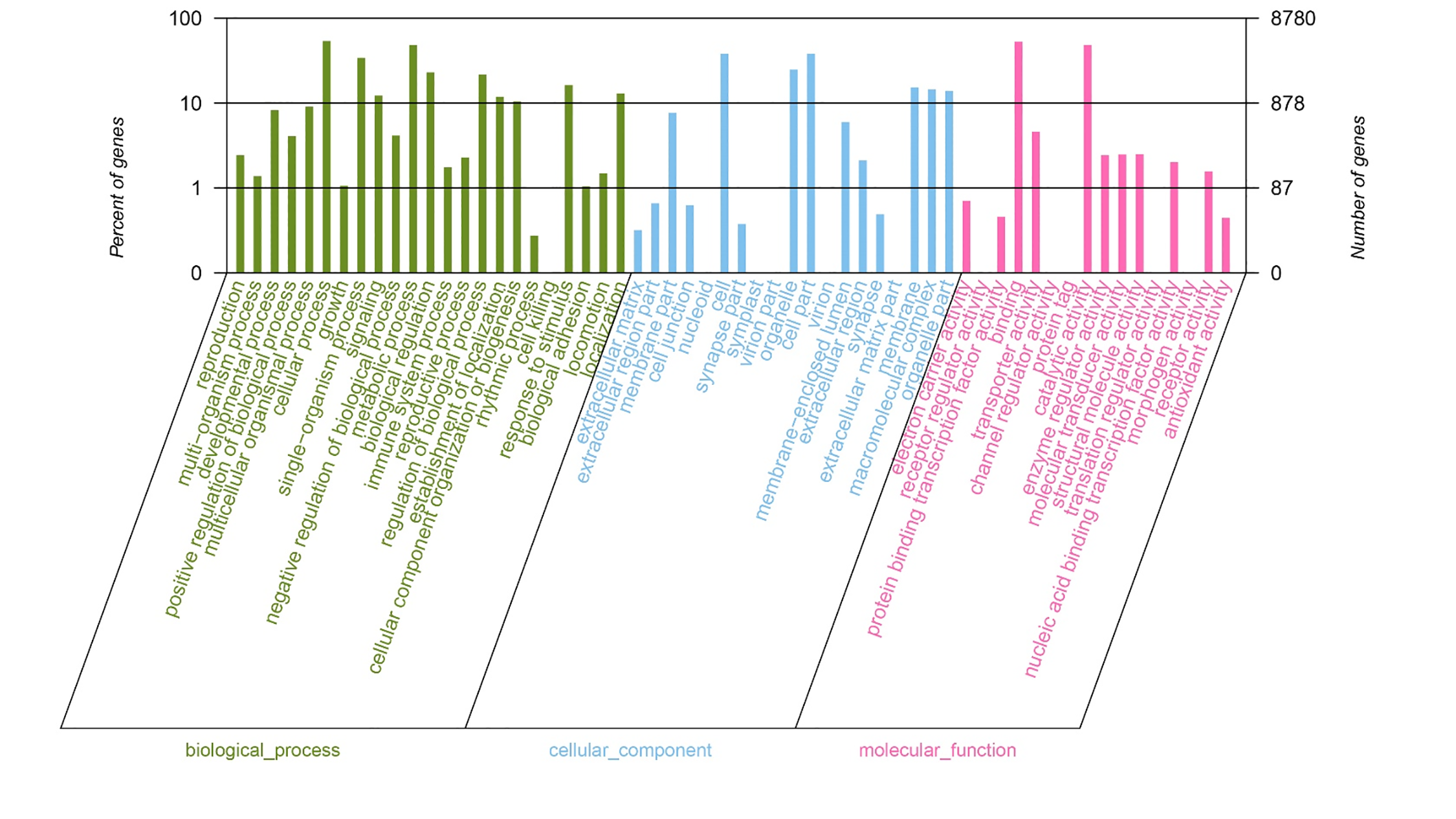

Supplement: S2 Fig — (TIF) [file pone.0131503.s002.tif]

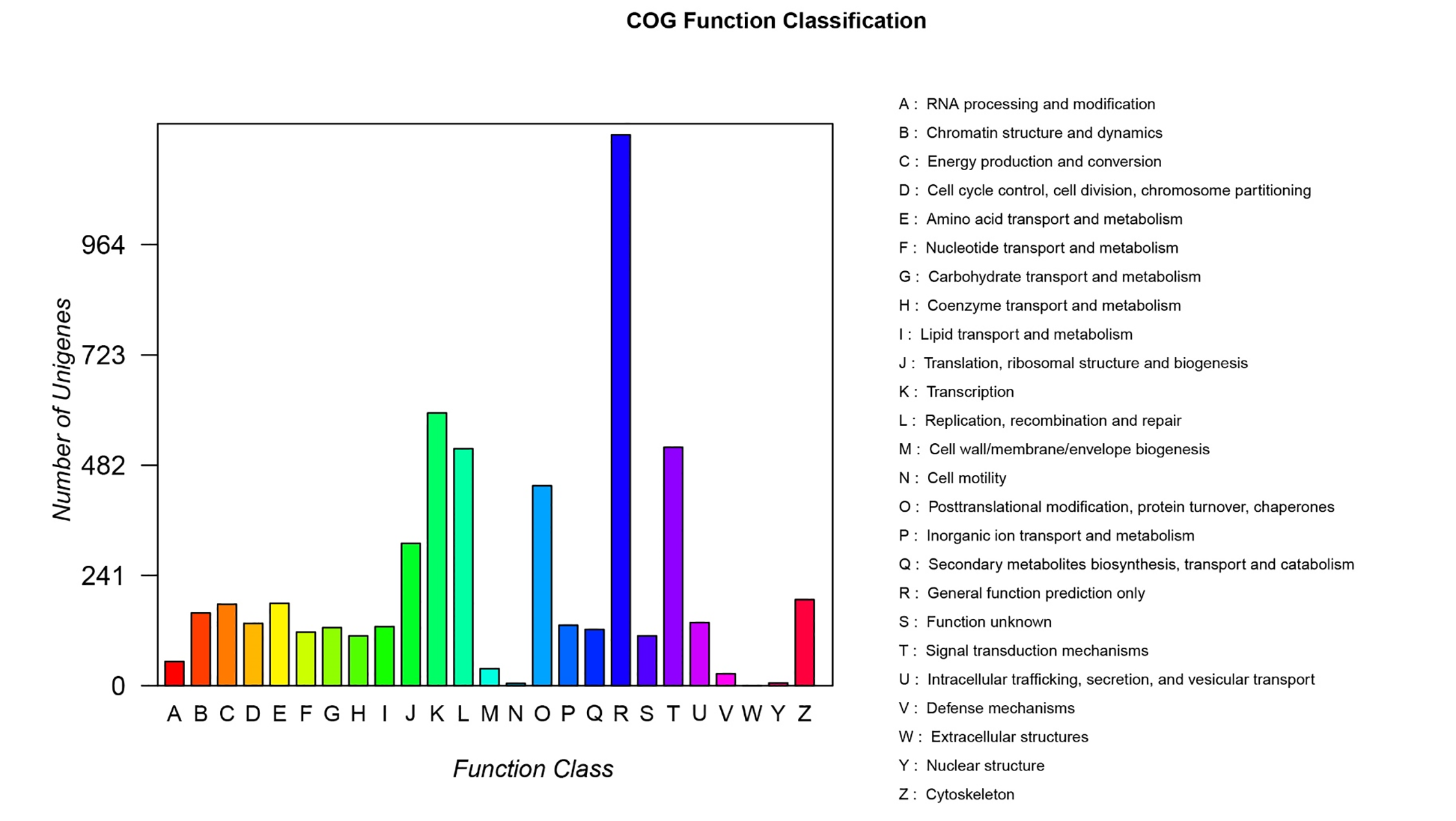

Supplement: S3 Fig — (TIF) [file pone.0131503.s003.tif]

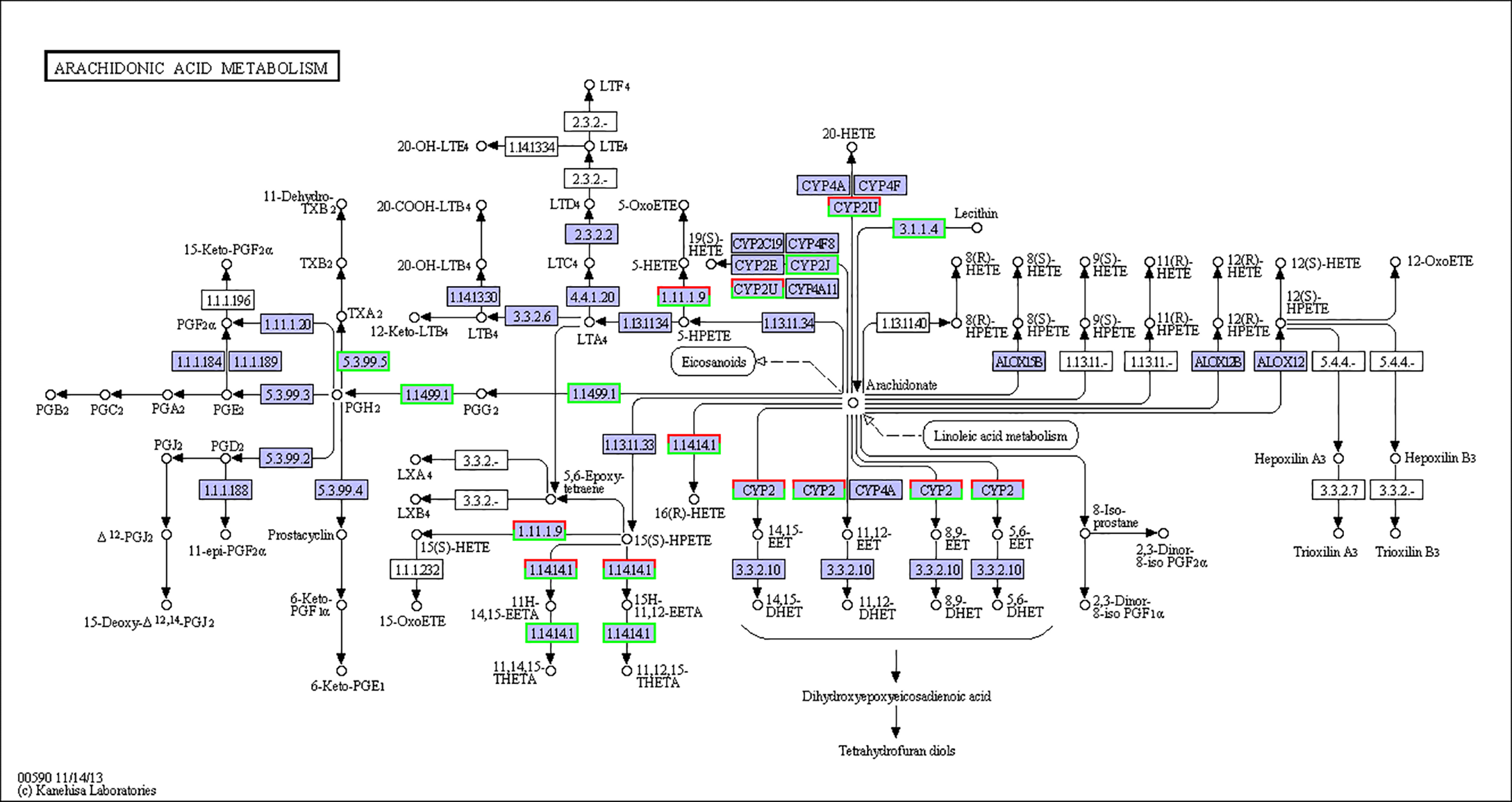

Supplement: S4 Fig — (TIF) [file pone.0131503.s004.tif]

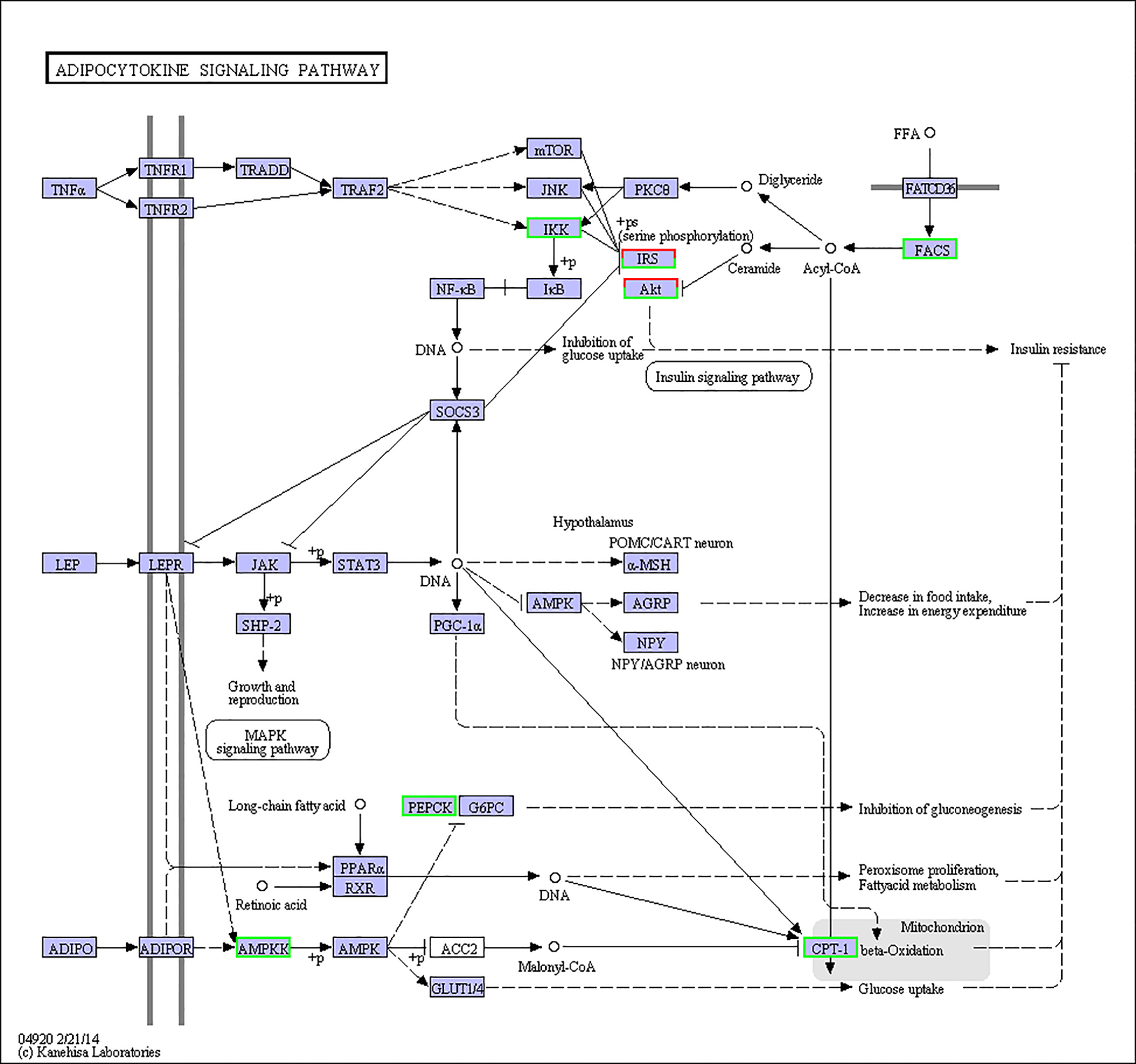

Supplement: S5 Fig — (TIF) [file pone.0131503.s005.tif]

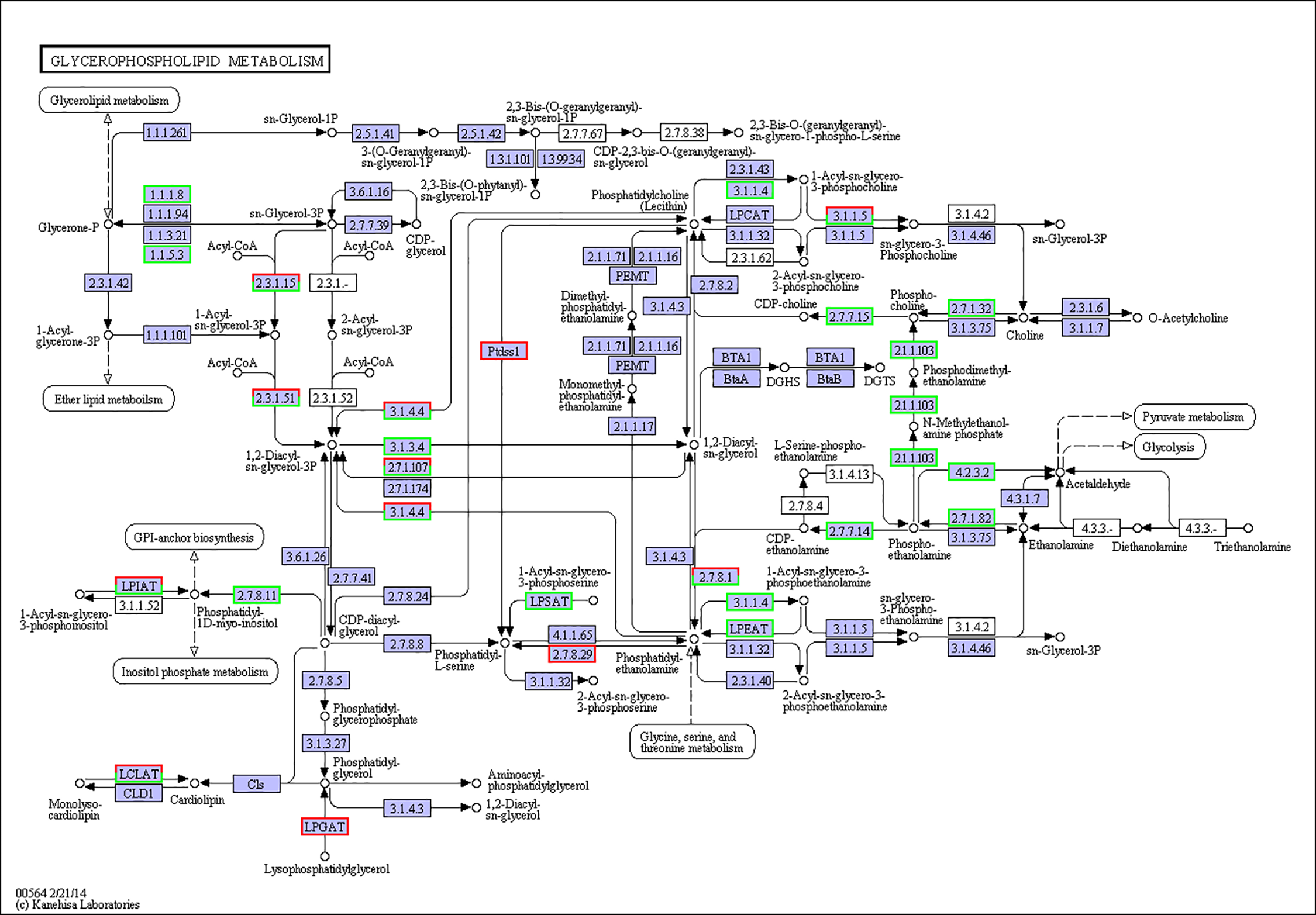

Supplement: S6 Fig — (TIF) [file pone.0131503.s006.tif]

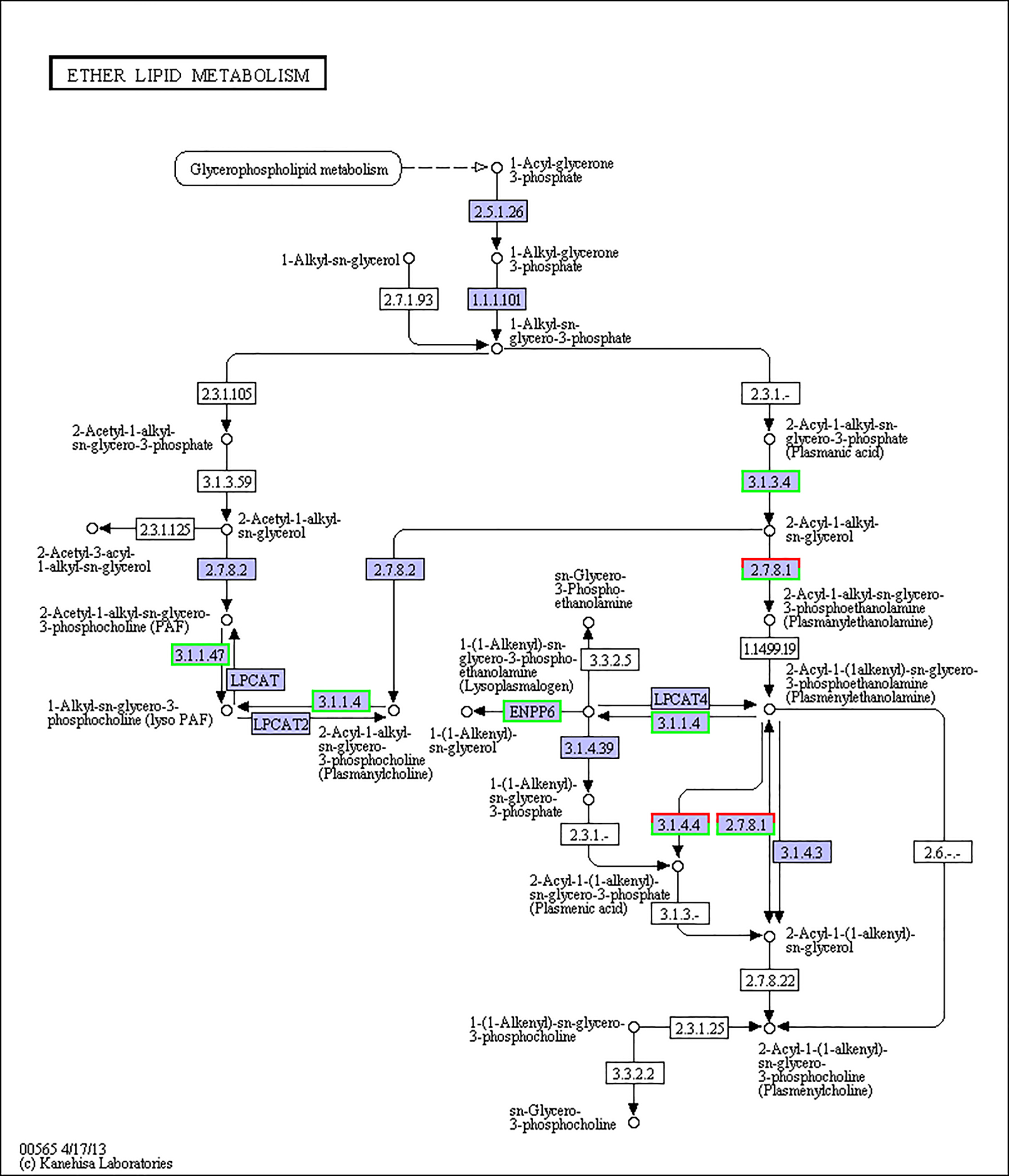

Supplement: S7 Fig — (TIF) [file pone.0131503.s007.tif]

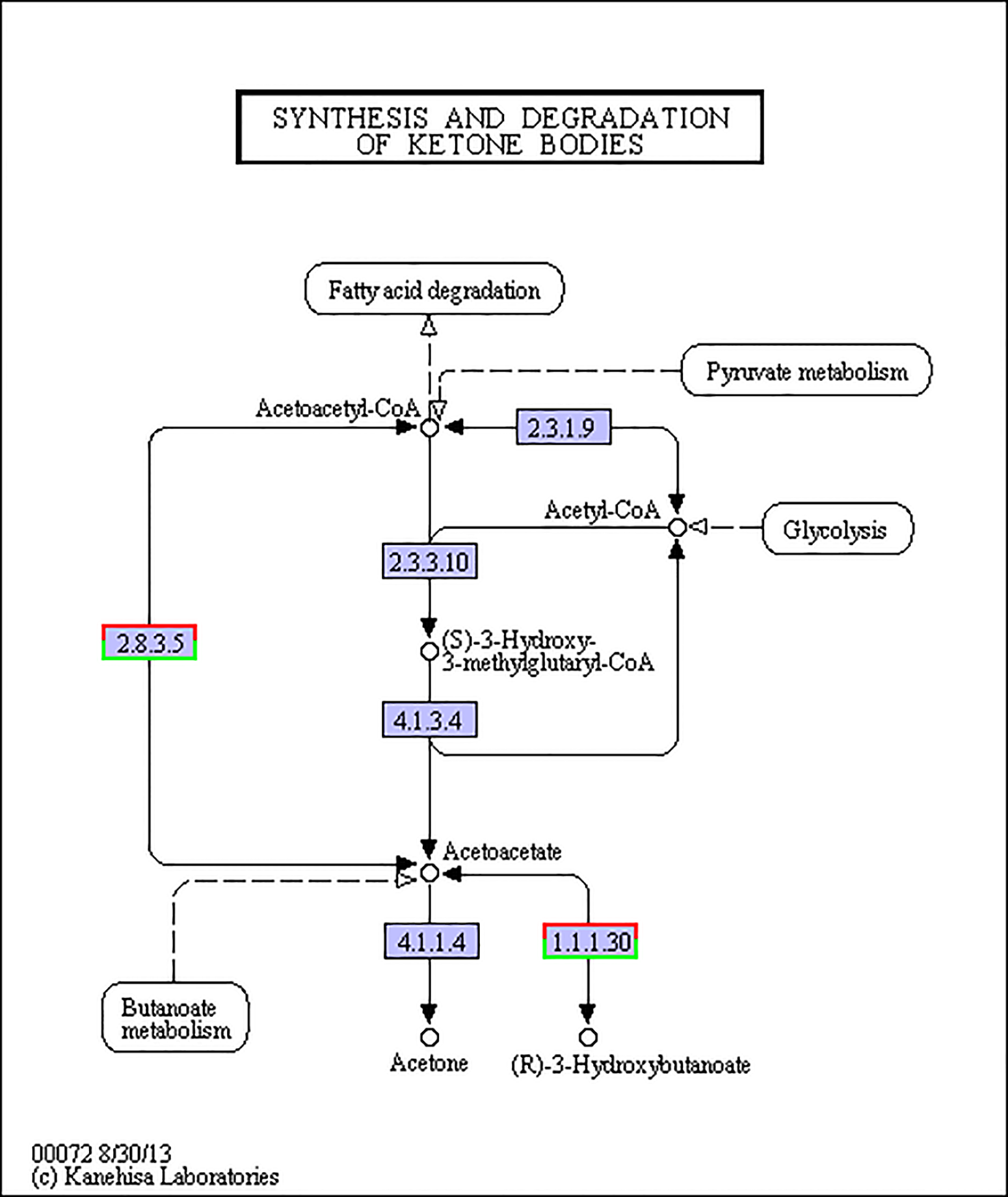

Supplement: S8 Fig — (TIF) [file pone.0131503.s008.tif]
